# Marine food web perspective to fisheries‐induced evolution

**DOI:** 10.1111/eva.13259

**Published:** 2021-06-24

**Authors:** Sara Hočevar, Anna Kuparinen

**Affiliations:** ^1^ Department of Biological and Environmental Science University of Jyväskylä Jyväskylä Finland

**Keywords:** eco‐evolutionary change, fisheries‐induced evolution, life history, marine food webs, recovery potential, size‐selective fishing, trophic cascades

## Abstract

Fisheries exploitation can cause genetic changes in heritable traits of targeted stocks. The direction of selective pressure forced by harvest acts typically in reverse to natural selection and selects for explicit life histories, usually for younger and smaller spawners with deprived spawning potential. While the consequences that such selection might have on the population dynamics of a single species are well emphasized, we are just beginning to perceive the variety and severity of its propagating effects within the entire marine food webs and ecosystems. Here, we highlight the potential pathways in which fisheries‐induced evolution, driven by size‐selective fishing, might resonate through globally connected systems. We look at: (i) how a size truncation may induce shifts in ecological niches of harvested species, (ii) how a changed maturation schedule might affect the spawning potential and biomass flow, (iii) how changes in life histories can initiate trophic cascades, (iv) how the role of apex predators may be shifting and (v) whether fisheries‐induced evolution could codrive species to depletion and biodiversity loss. Globally increasing effective fishing effort and the uncertain reversibility of eco‐evolutionary change induced by fisheries necessitate further research, discussion and precautionary action considering the impacts of fisheries‐induced evolution within marine food webs.

## 1 INTRODUCTION

Human exploitation of marine resources dates back to a prehistoric era. Excavations from Vanguard and Gorham's coastal caves by Gibraltar convey the appetite of our closest relatives, Neanderthals, whose diet included marine mammals, seafood and fish (Stringer et al., 2008). The exploitation intensity has extensively amplified since, and the human population became much reliant on environmental, economic and social benefits sustained by marine ecosystems (Konar et al., 2019). Increased fishing pressure has been ensuring livelihood to a quarter of a billion people and has contributed substantially to the global economy (Teh & Sumaila, 2013). Yet, applied fishing methods have not been sustainable. A majority of commercially fished stocks are considered overfished (FAO, 2020), and effective catch per unit of effort is decreasing despite that the size of the fishing fleet has doubled since the 1950s (Rousseau et al., 2019). The future could still be optimistic as the status of global fish stocks is not homogenous but rather considerably varying among fisheries and locations, demonstrating the influence of successful management strategies (Hilborn et al., 2021). The general trend implies that while poorly managed stocks continue to decline, the biomass of well‐managed stocks is in stabilising or rebuilding state (Worm & Branch, 2012). Nevertheless, notwithstanding the intensity of management implementations, some restrictions have still proved insufficient by not accounting for the interlinking effects of biotic and abiotic environmental conditions, nor for the possibility of regime shits (Perälä et al., 2020; Shelton et al., 2006).

Long‐term exposure to intense and selective fishing does not only alter the species composition and deplete their biomasses but can also incite a genetic change in heritable traits (Law, 2007). The selective evolution can be attributed to fisheries when heritable trait shifts of exploited fish stocks are a consequence of underlying genetic changes induced directly by fishing activity and thus not driven by the environmental nor trophic drivers (Kuparinen & Merilä, 2007). Although heavily harvested stocks often show trajectories towards ‘live fast, die young’, fishing can induce selection on different traits, depending on fishing strategy and gear. Phenotypic plasticity (Kuparinen & Merilä, 2007), genetic swamping (Pukk et al., 2013) and demographic effects, such as genetic drift (Kuparinen & Hutchings, 2017), can also trigger similar trajectories as fisheries‐induced evolution (FIE), making FIE difficult to recognize (Heino & Dieckmann, 2009; Laugen et al., 2014). Moreover, the genetic architecture of a trait exposed to fishing mortality can also affect the extent of eco‐evolutionary changes in harvested species (Kuparinen & Hutchings, 2017).

In the last decades, the evidence on FIE has been presented and challenged several times (Andersen & Brander, 2009; Browman et al., 2008; Jørgensen et al., 2007; Kuparinen & Merilä, 2007; Pinsky et al., 2021), ever since the seminal study on Arcto‐Norwegian cod, demonstrating the selective force of age‐specific harvest (Law & Grey, 1989). Notwithstanding remaining debates and challenges, to this day, a mounting body of literature indicates that FIE is supported not only theoretically but also empirically (Alós et al., 2014; Jakobsdóttir et al., 2011; Uusi‐Heikkilä et al., 2015; Young et al., 2020). However, while we are becoming more familiar with the consequences that FIE can have on population dynamics of a single species (Dunlop et al., 2015; Enberg et al., 2010; Hollins et al., 2018; Hutchings & Fraser, 2008; Kuparinen & Merilä, 2007), we are just beginning to grasp the array and intensity of its far‐reaching effects on the dynamics of the entire marine food webs.

Human‐mediated removal of selected individuals within a population can change the structure of a food web (e.g. Daskalov et al., 2007). The consequence of fishing can be seen as altered predation regimes which can cascade down the food web and result in eco‐evolutionary changes at lower, nontargeted species (Perälä & Kuparinen, 2020; Wood et al., 2018). Given the FIE has the potential to alter biological traits of species or their life‐history stages significantly, the induced changes could manifest in a modified ecological role of species under harvest, their trophic position and interactions (Audzijonyte et al., 2013). Moreover, since marine food webs are globally connected (Albouy et al., 2019), the generated eco‐evolutionary changes in species traits within the food web may have the potential to widely propagate across the ecosystems and modify their functioning and, ultimately, services and nature's contribution to people (Díaz et al., 2018).

**FIGURE 1:**
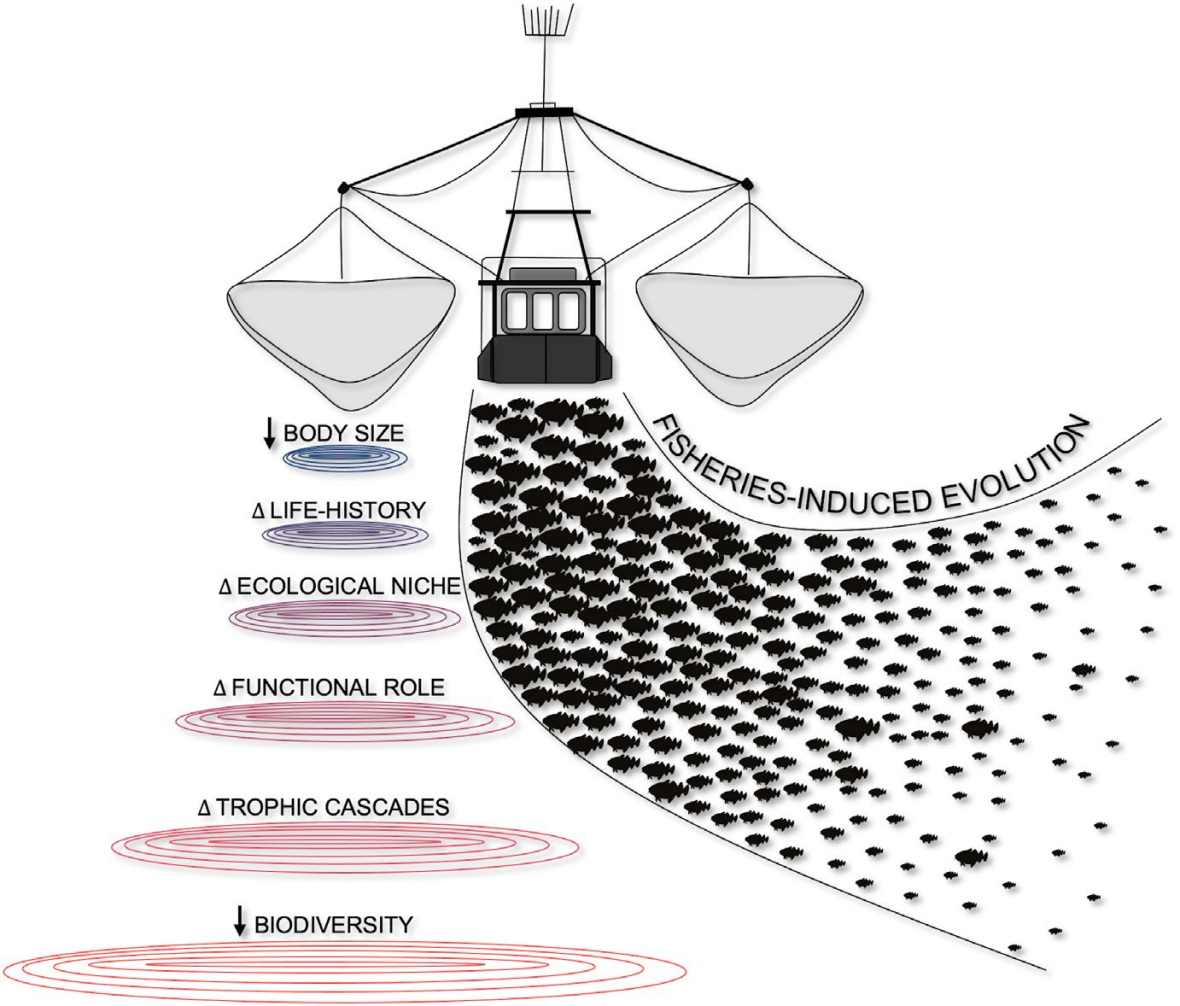
Fisheries‐induced evolution resonating through different levels of biological organization within the marine food web. Starting with changes in body size and life‐history traits at the individual level, moving to altered ecological and functional role at species level, to trophic cascades and biodiversity loss at the community level. The illustration is a simplified summary of the main mechanisms discussed in this perspective

In this perspective, we look at the current status of scientific knowledge on the influence of FIE within marine food webs (Figure 1). Although only a minority of the findings that follow stems explicitly from studies that have succeeded to detect FIE, the perceived mechanisms are alike and, therefore, instructive to each other (Hutchings & Kuparinen, 2021). Fishing impacts that deprive population genetic diversity or induce a direct demographic truncation (Kuparinen et al., 2016) by size‐selective biomass removal can be ecologically analogous to evolutionary shifts towards smaller body size. Bearing that in mind, we briefly explore five pathways through which FIE, driven by size‐selective fishing, might influence the intrinsic structure and functioning of marine food webs. We highlight (i) how FIE in physiological, behavioural and life‐history traits might shift the ecological niche of harvested species, (ii) whether altered maturation schedule and stock productivity could impact biomass flow, (iii) how modified life histories can trigger trophic cascades and (iv) how the declining body size of apex predators may modify their functional role. Finally, we look at (v) the weakened recovery potential of depleted stocks and how it may accelerate biodiversity loss.

## 2 BODY SIZE AND SHIFTING ECOLOGICAL NICHE

Nonrandom fishing exploitation can exert a selective pressure and induce evolutionary changes in physiological, behavioural and life‐history traits of fished stocks (Diaz Pauli & Sih, 2017; Jørgensen et al., 2007; Kuparinen & Festa‐Bianchet, 2017; Olsen et al., 2004; Uusi‐Heikkilä et al., 2015). The most common driver of FIE is a long‐term size‐selective harvest under which the population endures altered rate, probability and timing of fishing mortality. Although this fishing type has been predominantly studied, FIE does not require fishing to be necessary size selective (Biro & Post, 2008). Size‐regulated management applications in commercially harvested stocks and observed life‐history changes that are measurable make size‐selective driver of FIE simpler to explore. For example, the costs of size‐selecting harvest are maximized short‐term benefits, which can be seen in selection for slower or faster growth rates, or truncated age and size distribution (Hsieh et al., 2006; Jørgensen et al., 2009; Kindsvater & Palkovacs, 2017). Fishing season, location, gear limits or minimum landing sizes expose individuals that fall under these fishing bounds to fishing mortality, which generally exceeds natural mortality and has the opposite trend; the vulnerability to fishing increases with the body size of an individual (Hansen et al., 2011).

Body size is a central biological trait that correlates strongly to several life‐history and behavioural traits (e.g. Walsh et al., 2006) and portrays the underlying characteristics of food webs (Brose et al., 2006; Trebilco et al., 2013; Woodward et al., 2005). The size continuum, where larger individuals eat smaller ones, is typical of marine food webs. The diet portfolio (i.e. number of available prey taxa) of marine species is closely size‐related and can become more diverse with increasing body size of consumers (Nordström et al., 2015). As the diet expands, the species becomes more generalist by preying upon a broader range of prey species, allowing it to be disproportionally less dependent on a single prey species. While the extent of the influence that size has on the diet composition differs among fishes (Barbini et al., 2018; Schafer et al., 2002), the general trends show a positive correlation between body size and trophic niche width. Thus, as the fisheries select against large adult body size, the dietary range of harvested species may become narrower and its risk‐spreading effect less significant. This could reduce the resilience of harvested species to prey fluctuations or to increased intraspecific competition for resources (Jacobson et al., 2018). Simultaneously, fisheries‐induced changes in the size distribution could reshuffle predator–prey interactions or decrease the number of feeding links, and thus narrow the available range for diet switching among alternative prey species. Loss of feeding links could result in an impoverished trophic diversity (measured as the number of species or life‐history stages at different trophic levels) and ecosystem productivity (Poisot et al., 2013).

A small size truncation can significantly expose harvested species to a greater number of potential predators and considerably increase its natural mortality rate up to 50 per cent, while in other instances, predation rate can decline (Audzijonyte et al., 2013a). The decline may be due to smaller individuals being predated upon less or due to a behavioural shift in predator avoidance, where in order to escape predator pressure, harvested populations with a reduced size structure relocate to a safer areas or refugia (Audzijonyte, Kuparinen, & Fulton, 2013). The process could be reversely compared with ontogenetic shifts in movements and habitat use (Reis‐Filho et al., 2019) where younger and smaller individuals tend to cluster in less exposed areas with milder competition for space and resources, resulting in a lower success rate of predator attacks (Van De Wolfshaar et al., 2006).

Shifts in spatial distribution may also occur due to plastic or evolutionary changes in behavioural traits of harvested stocks (Diaz Pauli & Sih, 2017). Depending on the type of fisheries and fishing gear (i.e. passive such as longlines or active such as trawling), fisheries can promote a certain type of behaviour (i.e. shy or bold) (Arlinghaus et al., 2017; Diaz Pauli & Heino, 2014; Diaz Pauli & Sih, 2017; Kuparinen et al., 2009; Uusi‐Heikkilä et al., 2008). Because these behavioural traits tend to be at least phenotypically correlated with body size, bolder individuals could be inevitably selected against more frequently, irrespective of fishing gear (Biro & Post, 2008). Fast‐growing fish that tend to be more active or bolder have higher metabolic demands and need to spend more time on foraging, which increases their vulnerability to fishing. Some harvested stocks with plausible fisheries‐induced changes in behavioural patterns can be seen to dive deeper (Handegard et al., 2003), to relocate their spawning grounds to habitats that are less accessible to fishing gears (Opdal & Jørgensen, 2015) or, as fishing gear selectivity experiment has shown (Özbilgin & Glass, 2004), to learn how to escape from fishing meshes (i.e. developmental plasticity).

Altered behavioural patterns and truncated size structure can influence interspecies interactions through which energy and resources are transferred between trophic levels (Trebilco et al., 2013). Body size tends to correlate with higher trophic position positively and with the abundance negatively, directing the energy flow from abundant, smaller body sized nodes (i.e. species or life‐history stages of species) to less abundant, larger body sized nodes (Jacob et al., 2011; Nordström et al., 2015). These properties form the basis of the size spectrum models recently applied to analyse, for example, fisheries‐induced changes in the ecosystems (Jennings & Blanchard, 2004). Size hierarchy in predator–prey interactions constitutes the food‐web topology and improves its stability (Blanchard et al., 2011; Riede et al., 2011). Moreover, because body size correlates with life history, the predator–prey size ratio could be affected by fisheries‐induced changes in growth rate, determining the time an individual spends in each size class.

## 3 MATURATION SCHEDULE AND BIOMASS FLOW

The most typically observed aftermath of overexploiting harvesting practices is the tendency to select for earlier maturation age in fish (Hutchings, 2005; Olsen et al., 2004; Swain, 2011). Traits related to maturation timing can change faster than other life‐history traits, which may contribute to their susceptibility to FIE (Audzijonyte et al., 2013b). Although the trend of maturing at earlier age tends to be associated with a decrease in stock abundance and a release of density‐dependent processes that allow for a faster somatic growth rate (Roff, 2002), selection experiments have shown that fishing can also be a driving force of phenotypic and genotypic change in heavily exploited stocks (reviewed by Diaz Pauli & Heino, 2014). Intensive fishing mortality and selective removal of larger and often late‐maturing individuals can change the allelic frequency of the inherited trait in a population. This can consequently increase the probability that genetic encoding, favoured by fishing, will be passed to the next generation. For example, 37 commercially harvested fish stocks mainly inhabiting marine food webs of temperate regions showed evolutionary changes in maturation schedule and probabilistic maturation reaction norms, both highly correlated with fishing intensity (Sharpe & Hendry, 2009).

The maturation schedule is often a proximate determinant of individual fitness, including age‐related survival probability and reproductive output (Hutchings, 2005). Individual fitness can translate to a stock reproductive potential based on the combined effects of population abundance, sex ratio, age and size structure (Morgan & Brattey, 2005). Therefore, by selecting for earlier‐maturing life histories, size‐selective harvest can exert a directional pressure on an individual's energy allocation invested into reproductive and somatic growth and can, thereby, affect the stock productivity (Ohlberger et al., 2020). Paradoxically, a selection for life histories adapted to fishing exploitation can lead to depleted reproductive potential. Smaller female spawners tend to produce fewer and less viable eggs, which decreases an individual's lifetime fecundity, population's total egg production and stock's productivity (Birkeland & Dayton, 2005; Ohlberger et al., 2020; Walsh et al., 2006), resulting in a reduced yield and economic losses (Laugen et al., 2014). We might be underestimating the possible aftereffects of FIE on the biomass increase and variation within trophic networks (Jennings & Blanchard, 2004), given the influx of recruits is often disproportionately larger among bigger and older females compared with smaller and younger ones (Barneche et al., 2018).

Size diversity of fish life‐history stages can notably influence the biomass flow and increase food‐web stability (Bland et al., 2019). Indeterminate somatic growth is typical among fish, meaning that age and body size correlate closely (von Bertalanffy, 1938). Therefore, the consequences of fisheries‐induced changes that select for earlier maturation at smaller body size have the potential to destabilize the food‐web functioning by truncating the age or size structure of the harvested population (Table 1). Such food webs can endure higher vulnerability to environmental changes and intensify fishing mortality unless the fishing effort is relaxed (Brose et al., 2006; Kuparinen et al., 2016). A simulation study of a food‐web model empirically parameterized for the Lake Constance ecosystem showed that by decreasing the body size ratio between the feeding links, fishing increases the biomass fluctuation of harvested species and co‐existing species in the food web (Kuparinen et al., 2016). The magnitude of fluctuations became especially severe and displayed a longer‐lasting effect when evolutionary changes due to selective removal of old and large individuals were heritable and considered within the food‐web architecture. Although the harvested fish species have recovered substantially once the simulated fishing was ceased, the population's recovery had not reached its prefishing values when traits were heritable. These model predictions illustrate the indispensable impact of a selective harvest, inflicted directly on the population dynamics of harvested species (Anderson et al., 2008; Hsieh et al., 2006) and implicitly on the dynamics of the ecosystem (Kuparinen et al., 2016; Perälä & Kuparinen, 2020).

**TABLE 1:** Fisheries‐induced evolution in marine food webs. Potential pathways through which fisheries‐induced evolution may alter the structure and functioning of marine food webs

Potential driver of FIE	Individual consequence	Population consequences	Food‐web consequence	Challenges
Higher mortality of a particular genotype	Compromised genetic diversity	Population may become less resilient or adaptable to environmental changes and regime shifts	The probability of missing links increases, and the food web may pass the tipping point of trophic cascades	What is the tipping point at which the fisheries‐induced changes in intraspecies interactions translate to interspecies interactions? How can this tipping point be identified or predicted?
Higher fishing mortality of larger individuals	Changes in somatic growth rate, smaller body size, smaller differences in body size among life‐history stages	Truncated size structure and size diversity of life histories can magnify the fluctuations in abundance of the fished population due to higher intraspecific competition for resources and more potential predators	The reduced body size ratio among existing feeding links can further decrease the food‐web stability by increasing the biomass fluctuations among links	Can fisheries‐induced changes in growth rate shape the growth rates of their feeding links and thus alter the functionality of marine food webs?
Higher fishing mortality of older individuals	Earlier maturation at smaller body size, higher maternal costs, reduced reproductive output and risk‐spreading effect	Low recruitment biomass that is released during a shorter spawning window may result in more frequent mismatch occasions, and a weaker, longer and more uncertain population recovery potential	Strong fluctuations in the biomass of recruits could destabilize the trophic flow in the food web and reduce its buffering capacity to environmental variability	Could fisheries‐induced changes in altered maturation schedule reduce the temporal and spatial synchrony in ecological efficiency of feeding links? To what extend could mismatch events affect the stability of marine food webs?
Higher fishing mortality of bolder individuals	Gear avoidance, timid individuals, decreased foraging efficiency, shorter spawning migrations, reduced parental care	Population with a prevalent proportion of timid behavioural type may have compromised metabolic rate and reproductive success	Timid individuals might connect to a lower number of trophic links and thus reduce food‐web connectivity and robustness	How can FIE in traits that influence the metabolic rate impact the trophic efficiency of marine food webs?
Higher fishing mortality in the oldest life‐history stages of apex predators	Vulnerability to predation, ontogenetic changes in diet and narrower diet portfolio	Increased natural mortality rate and altered functional role	With downsized life histories, the number of feeding links may decline along with prey switching and predator control and shift the control to species at lower trophic levels	Could marine food webs become more prone to extinction if the diet niche of apex predators narrows with downsized life‐history stages?

## 4 ALTERED LIFE HISTORIES AND TROPHIC CASCADES

Stocks can be regulated by the availability of resources and the consumers’ presence. Both restrains intertie the role of the stock within the food web, which makes it susceptible to trophic cascades. Intense harvest has a potential to change the strength of the trophic cascades on a short scale due to ecologically induced changes arising from the removal of fish biomass (Altieri et al., 2012; Mumby et al., 2006) or on a long scale due to evolutionary‐induced life‐history changes (Audzijonyte, Kuparinen, & Fulton, 2013; De Roos et al., 2006; Kuparinen et al., 2016; Perälä & Kuparinen, 2020; Start, 2018; Wood et al., 2018). By definition, trophic cascades entail the changes in abundance or biomass density of a functional group or population to propagate beyond one trophic link (Pace et al., 1999; Paine, 1966). An example can be the top‐down cascades in the food web of Scotian Shelf that were induced by overfishing of Atlantic cod and other large predators (Scheffer et al., 2005). The absence of the top predators influenced the community structure and competitive interactions. Predation and competition release increased the abundance of small pelagic fishes and benthic invertebrates, which reduced the abundance of larger zooplankton species and reduced grazing pressure on phytoplankton, leading to lower levels of nutrients. Similar ecological cascading mechanisms, triggered by harvesting, have been reported worldwide, from simpler food webs in reef communities (Mumby et al., 2006), kelp forest (Tegner & Dayton, 2000) and saltmarsh ecosystems (Altieri et al., 2012), to complex ecosystems in the open ocean (Frank et al., 2005).

Ecological changes that cascade through the food webs can also generate an adaptive response in species that are not directly targeted by fishing. Re‐balanced abundance and biomass open pathway for evolutionary changes arising in traits that are influenced by density‐dependent processes. For instance, a modelling study tracking the evolution of a genotypic‐based competition–defence trade‐off showed that the eco‐evolutionary response to fishing could cascade downward to nontargeted species (Wood et al., 2018). The two underlying mechanisms driving the cascades were feeding availability and predator vulnerability. The food web constituted of four trophic levels. As the harvest of top predators started, the food web showed typical top‐down cascade: the abundance of top predators declined, leading to increase in secondary consumers, decrease in primary consumers and increase in producers. Simultaneously, the tracking of the competition–defence trade‐off showed that the impact of FIE and its direction alternated among trophic levels in a corresponding manner alike to the ecological top‐down cascade. This pattern highlights that fishing can generate ecological and evolutionary cascades just through the biomass removal due to changes in prey density and predation regime.

FIE in life‐history traits of fished species may lead to trophic cascades as well. The maturation schedule is a strong contingent of reproductive success and population dynamics. This connection essentially provokes the assumption that depleted stocks with altered spawning potential and higher recruitment variability may make the food web more sensitive to top‐down or bottom‐up cascades. For example, the reduced disparity between the life‐history stages of harvested species undergoing ontogenetic shifts could increase the potential for mismatch events (Cushing, 1990). Maturation at bigger body size in broadcast spawners is often positively related to the duration of spawning season and the number of spawning events (Kjesbu et al., 1996), which leads to greater chances of finding favourable conditions for larval survival (James et al., 2003). Therefore, a truncated size diversity of life‐history stages may lower the likelihood of newly hatched larvae overlapping in time and space with the peak of its prey availability or quality. The consequences could be reduced recruitment success and increased temporal fluctuations in the abundance of harvested stocks (Hsieh et al., 2006; Siddon et al., 2013), which could feedback ecological cascades.

To quantify the impact that fisheries‐induced changes in life‐history evolution may have on the aquatic food webs, Perälä and Kuparinen (2020) extended the allometric trophic network and allowed two life‐history traits of adult fished stocks to evolve with time. They simulated small and large size‐selective fishing of perch in Lake Constance and tracked how the evolutionary changes in the asymptotic body size and reproductive costs develop within each of the five life‐history stages (larvae, juveniles, and 2‐year, 3‐year and 4+ year adults). Findings showed that fishing type can influence the direction and the extent of evolutionary change in body size. Ecological and evolutionary changes driven by the large size‐selective fishing generated stronger biomass changes within the perch life stages and within the food web compared with small size‐selective fishing. Key mechanisms behind this were changes in metabolic rates and maintenance costs of harvested individuals along with their body size changes, as well as changes in the maintenance costs due to changes in the timing of maturity, that is the survival costs of reproduction (Bell, 1980). Therefore, populations exposed to large size‐selective fishing had more truncated body size, which has contributed to the increased feeding rate of younger life stages and predatory pressure of their prey (including fish larvae).

Interestingly, the presence of evolution seems to have played an especially strong role for the ecological cascades in the food web under small size‐selective fishing, where the ecological changes in biomasses of some functional groups would be hardly observable otherwise (Perälä & Kuparinen, 2020). This counterintuitive outcome could be an artefact of the model's assumptions and limitations. For example, the modelling approach has not fully emulated the mechanisms of a food web as perch was the only species susceptible to evolutionary changes, and the diet was not evolving along with body size, meaning that food‐web feedbacks may be quite conservative. Findings emphasize that recognising the origin and predicting the outcomes of coupled eco‐evolutionary cascades can be challenging.

Trophic cascades can emerge from a complex interaction of biological mechanisms and human‐mediated drivers that can have amplifying or buffering effects. Multiple anthropogenic drivers can synergistically destabilize the system not just by the removal of feeding links but also by the introduction of new ones. For instance, the trophic cascades in the food web of the Black sea show ecological changes triggered by overfishing, eutrophication and the introduction of invasive species (Daskalov et al., 2007). The cascades generated by the overfishing of top predators propagated downwards by altering the predator–prey interactions, while the introduction of invasive ctenophore induced an upward cascade due to high predatory pressure on eggs and larvae of commercial stocks. Despite that trophic cascade can occur within the food webs independently of fishing exploitation, modelling and empirical evidence suggest that fishing at one or multiple levels can provoke or neutralize the motion of trophic cascades. The intensity of cascades can be highly context‐dependent and can vary on a regional or local scale with environmental conditions and community structure (Pinkerton & Bradford‐Grieve, 2014; Shears et al., 2008). While in some systems, fishing can be a limiting factor that triggers cascading effects in biomass or abundance directly through the community structure and functioning, in other instances, the induced fluctuations can be buffered through the fishing activity itself (Andersen & Pedersen, 2010).

## 5 FADING APEX PREDATORS

**FIGURE 2:**
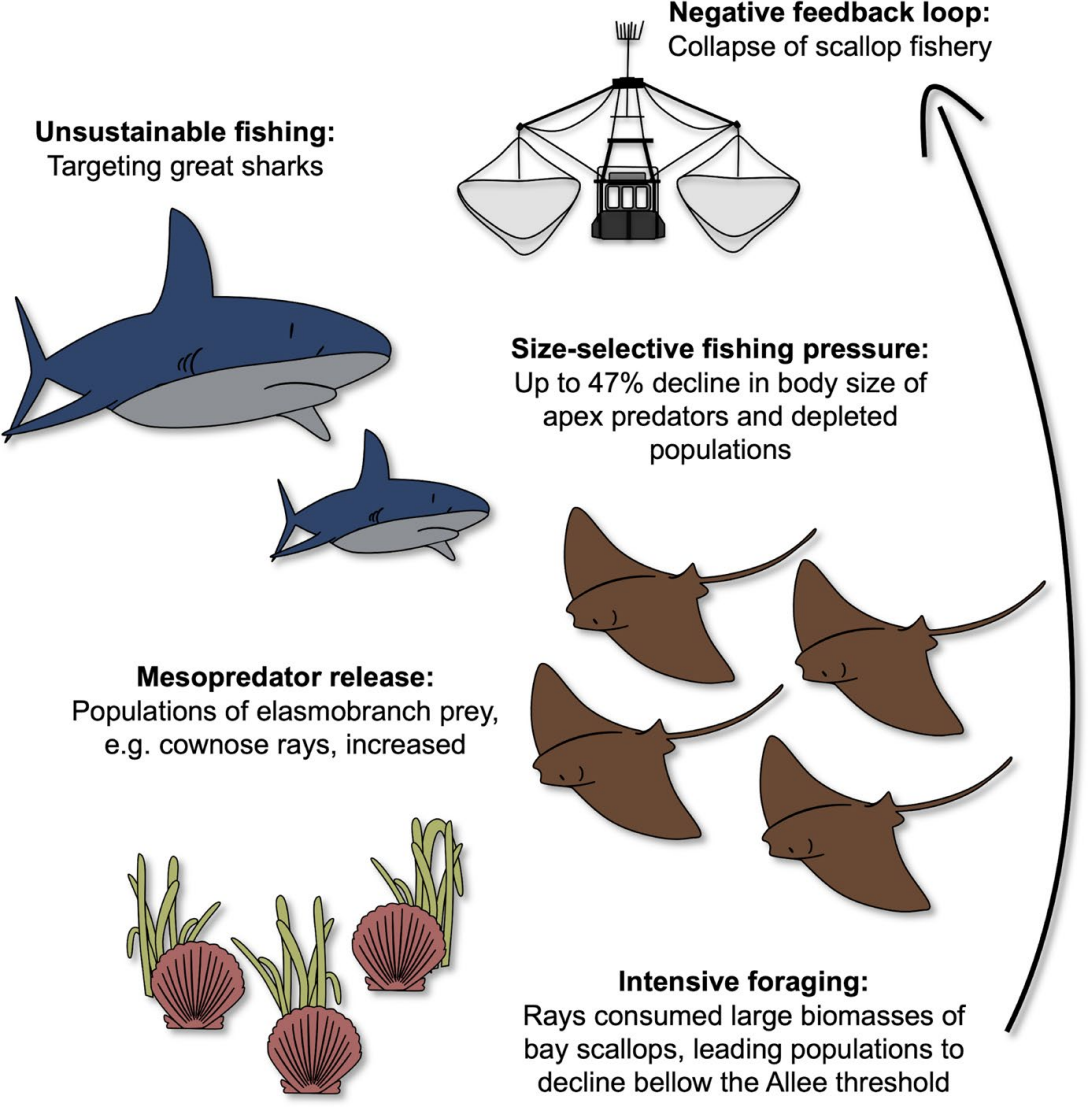
Example of cascading effects with a feedback loop generated by selective fishing of apex predators. Adopted based on Myers et al. (2007)

Apex predators are species at the highest trophic level of their ecosystem. Due to their large body size, the adults do not tend to have natural predators, and thus are not regulated by top‐down control but rather by prey‐availability (i.e. bottom‐up control) and self‐regulating processes (Wallach et al., 2015). Populations of apex predators across ecosystems have been under substantial declines, pushing several to the verge of functional extinction or pass it (Pauly et al., 1998; Ripple et al., 2014). Despite their low densities, apex predators are essential in maintaining the ecosystem in a balanced structure and healthy state through predation (Estes et al., 2011). Given this control, the overfishing of their populations can quickly cascade down the food web and increase the biomass of their prey species, usually mesopredators, which often tend to be species of fisheries interest. For instance, overexploitation of large sharks along the northwest Atlantic coast has severely depleted shark populations and truncated their size structure with up to 17–47% decline in body lengths (Myers et al., 2007). This eased control over their mesopredatory elasmobranch prey, including cownose ray (*Rhinoptera bonasus)*, which became exceptionally abundant (Figure 2). With increased ray population, the pressure for its prey, commercially harvested bay scallop (*Argopecten irradians*), increased, which has resulted in a collapse of scallop fishery (Myers et al., 2007).

While the diet of most fish species is gape‐limited, as they tend to swallow a whole prey at once, apex predators can also prey upon bigger species (e.g. Lucifora et al., 2009). During their ontogenetic dietary shifts, apex predators can predate on all trophic levels (Navia et al., 2016). Size‐related traits such as life histories, metabolic rate, diet, feeding and haunting behaviour, and habitat preferences constitute the functional role of apex predators that shape marine communities (Heithaus et al., 2008). Given their functional role within the food web, apex predators tend to exhibit high topological uniqueness (Navia et al., 2016), meaning that there are not many species of similar network position. Therefore, replacing their function effectively would be difficult (Prugh et al., 2009).

Losing apex predators from the food web is not a solitary risk. Their unique life histories also constitute trophic diversity. Although life histories might not always be directly interlinked to the trophic level, their loss can affect the energy and biomass flow from one trophic level to another (Bland et al., 2019). Shifting life histories could reduce not just phenotypic diversity but also functional diversity, and thus through altered food‐web structure impact the productivity of the system (Poisot et al., 2013). Downsized size structure of apex predators may increase predation risk to their earlier life stages, as the probability of being predated by more species would increase if the time spent at the earlier life stage is longer. Buffering capacity of a food web can decline if the trophic diversity is reduced and may amplify the sensitivity of a food web to environmental variability (Kuparinen et al., 2019). Trophic levels provide a buffering effect that dampens biomass variations with progressing levels. Therefore, if size‐selective fishing truncates life histories of apex predators, we might lose some of this protecting support that trophic and functional diversity ensure.

## 6 RECOVERY POTENTIAL AND BIODIVERSITY DECLINES

Species life history underlines its population dynamics and influences its response to exploitation rates and extinction risk (Jennings et al., 1999; Jusufovski & Kuparinen, 2020; Rowe & Hutchings, 2003). Depending on the type of fishing selectivity, modelling studies point that the rate and time of recovery in an evolutionary change in a selected trait are slow and not necessarily certain (De Roos et al., 2006; Perälä & Kuparinen, 2020). FIE may contribute to a delayed population recovery, lead to a weaker recovery or prevent full recovery of the heavily harvested stock by driving it beyond its stabilization threshold (Hutchings, 2015). The iconic case is the Northwest Atlantic cod fishery, where the spawning biomass had been depleted beyond 94%, resulting in ecological collapse (Hutchings & Myers, 1994). The stock has yet not reached its prefishing size structure nor abundance despite reinforced moratorium and management mitigations (Brander, 2007; Hutchings & Kuparinen, 2020).

**FIGURE 3:**
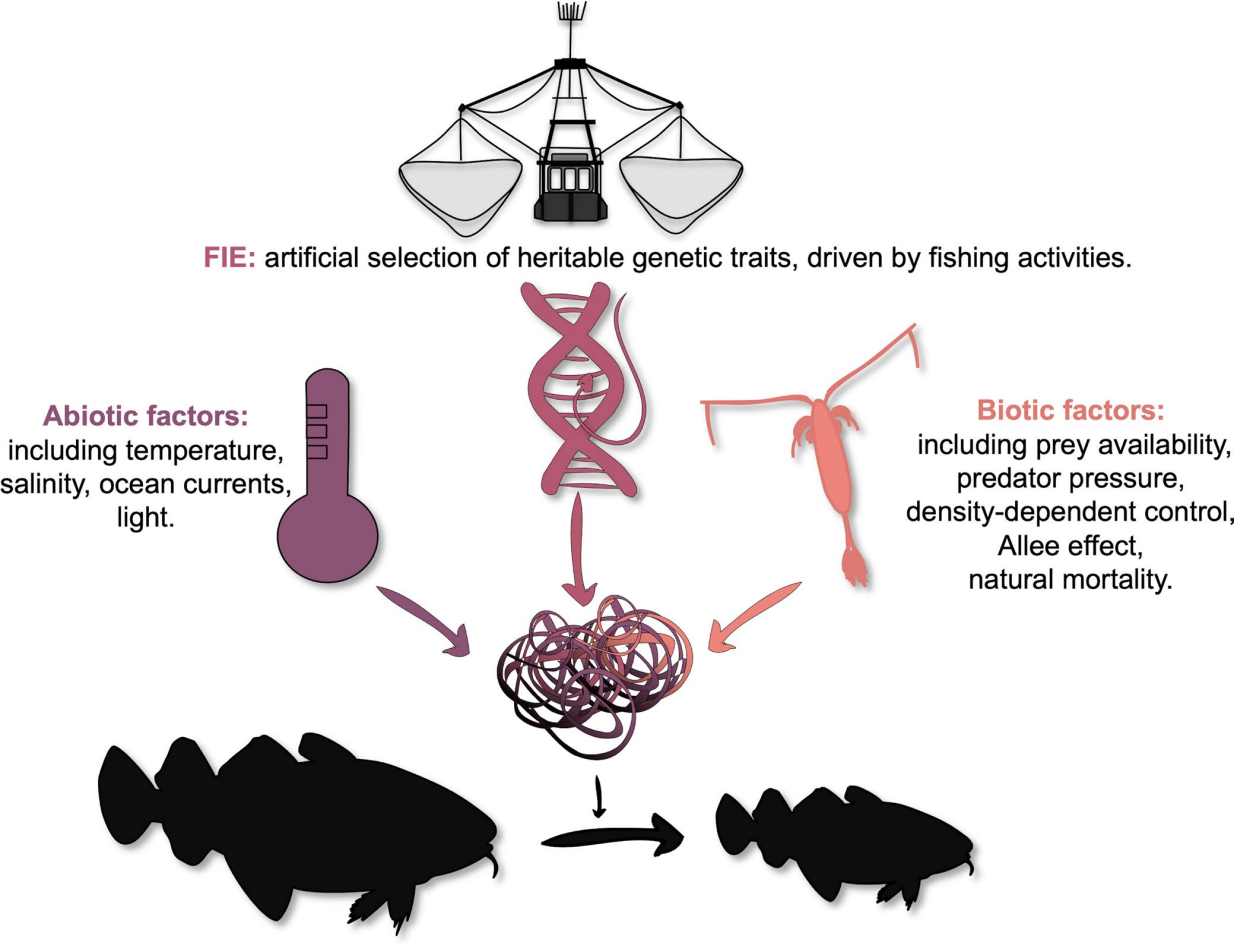
Schematic illustration depicting the complexity of identifying the extent of fisheries‐induced evolution apart from biotic and abiotic drivers

This case is critical as it points at the complexity of recognising the extent to which the FIE *per se* may have delayed the recovery (Figure 3) (Hutchings & Kuparinen, 2021; Pinsky et al., 2021). For instance, the increased natural mortality rates, environmental conditions, ongoing fishing activities and depensation effects have all influenced the lack of rebuilding (Shelton et al., 2006). Allee effect seems to have also been a significant contributor that has not just delayed or impaired the recovery period but has also greatly increased the uncertainty of recovery, as the population growth rate becomes lower and more variable at low abundance (Kuparinen et al., 2014). A meta‐analysis of 153 depleted marine stocks also elucidates how unpredictable can the recovery be (Neubauer et al., 2013). When analysed as separate effects, the historic intensity and duration of exploitation can shorten the recovery period (Neubauer et al., 2013). This outcome was suggested to be the aftermath of plastic and evolutionary changes in maturation schedule that may have increased the population growth rate when a population is exposed to long‐moderate or short‐intensive fishing. When the historic intensity and duration of exploitation were analysed as the interaction, the impact on the rebuilding of population biomass was negative and had prolonged the recovery time. The findings of this meta‐analysis are consistent with the modelling study from Lake Constance (Kuparinen et al., 2016), which showed that the rate or extent of recovery from eco‐evolutionary changes induced by FIE is not as fast nor as efficient as it is the recovery from ecological changes. Signs of severe depletion and collapsed fisheries can be seen from different parts of the globe (Myers & Worm, 2005; Neubauer et al., 2013; Sadovy de Mitcheson et al., 2013), and so are the consequences of overexploitation that have reduced species abundance and diversity in these depleted systems (Byrnes et al., 2007; Daskalov et al., 2007; Poisot et al., 2013; Rochet & Benoît, 2012; Thurstan & Roberts, 2010).

The diversity of species is declining, and fish species of larger body sizes are at high risk of extinction (FAO, 2020; Olden et al., 2007). Fishing activity can be deemed one of the main external drivers for extinctions within marine ecosystems, whether owing to direct overfishing or indirect habitat loss and fragmentation. Harvesting predatory species at higher trophic levels can destabilize trophic diversity and biomass flow within the food webs (Essington et al., 2006; Rochet & Benoît, 2012) and thus accelerate biodiversity loss. Predators promote the diversity of their prey in multiple ways, including through the active selection of the most abundant prey or dominant competitor, through the territorial distribution, consumer density and feeding rate (Koen‐Alonso & Yodzis, 2005; Ryabov et al., 2015). Selective fishing against larger body size can shift the trophic position of a predator, the size range of its prey, trophic efficiency, predation rate and density. Trophic generality (i.e. number of prey taxa in the diet) tends to positively correlate with body size, meaning that the number of species preyed upon by a predator can become lower if the predator's body size declines (Nordström et al., 2015). Moreover, reduced size diversity of life‐history stages can also compromise the robustness of predator's diet due to narrower ontogenetic diet shifts, which may otherwise secure a buffering impact for predator–prey dynamics and food‐web stability (Bland et al., 2019).

Beside trophic generality, trophic vulnerability (i.e. number of predator taxa) also tends to correlate with body size (Nordström et al., 2015). This correlation is negative and becomes especially relevant when the harvested species is a forage fish at the lower trophic level. Forage fish species tend to be well connected within the food web, and thus supporting the energy transfer from their resources to their predators. Therefore, fisheries‐induced evolutionary and ecological changes in the distribution of their size or life‐history stages can potentially impact the structure of a food web in two linear directions: through the altered upward or downward biomass flow. The robustness of a food web can also depend on the extent of trophic vulnerability of missing species. Some studies suggest that the extinction of the most vulnerable species that support many predator species tends to lead to the fastest collapse of the food web while the role of generality triggers less secondary extinctions (e.g. Jacob et al., 2011). Therefore, species (or life histories) that can decrease food‐web stability by going missing are not necessary only large species at the highest trophic levels but also small, highly linked species at lower trophic levels (Jacob et al., 2011; Navia et al., 2016).

Intertwined changes in declining body size, shifting diet, survival probability, spatial distribution and behavioural changes induced by overfishing can considerably modify the ecological role of species, alter the trophic structure of communities and reduce the value of fisheries catches (Essington et al., 2006). Although theoretical and empirical studies show that the recovery ability of a stock experiencing eco‐evolutionary changes due to overexploiting fishing practices can be substantially impaired and delayed (Eikeset et al., 2016; Hutchings & Kuparinen, 2020; Kuparinen et al., 2016; Kuparinen & Hutchings, 2012), these findings also indirectly suggest that a release of high fishing pressure holds the potential to rebuild the abundance of depleted species and to modify the food‐web structure yet again (Bieg & McCann, 2020; Ellingsen et al., 2020; Fung et al., 2013).

## 7 ANTHROPO‐PREDATOR WITH CONCLUDING REMARKS

Fisheries have become an integral part of marine food webs and have, along with climate‐induced changes, showed a considerable potential to modify the structure and function of ecosystems by gradually altering eco‐evolutionary processes (Audzijonyte, Kuparinen, & Fulton, 2013; Kuparinen et al., 2016; Pauly et al., 1998; Perälä & Kuparinen, 2020; Wood et al., 2018). Emerging evidence from diverse marine systems attests how through fishing ventures, the role of humans in ecosystem structure and functioning has intensified beyond, and unlike, one of the apex predators. We selectively strip the food webs of the individuals in a fully grown, reproductively most successful life stages at three times higher rate than the most voracious predators (Darimont et al., 2015). Doing so, we provoke faster phenotypic changes that are at least somewhat induced by size‐selective fishing (Audzijonyte, Kuparinen, Gorton, et al., 2013; Sharpe & Hendry, 2009).

Interestingly, although our trophic level within marine food webs is high, it does not exceed that of natural apex predators (Roopnarine, 2014). The reason could partially lie in the fishing‐down‐the‐food‐web strategy since due to depleted stocks at high trophic levels, fisheries shifts to stocks at lower trophic levels (Pauly et al., 1998). As we overfish authorized stocks, we extend our diet portfolio and move farther offshore, searching for new, yet unfished taxa (Pauly, 2020). The increased technological development has facilitated us to easily shift our ecological niche, quickly find prey, effectively avoid predators and, arguably, obviate top‐down, bottom‐up and density‐dependent control (Darimont et al., 2015; Wallach et al., 2015). These features distinguish us from apex predators and revolve us into sort of an»Anthropo‐predator«, as our impact on the ecosystems has notably amplified during Anthropocene, the epoch of human‐induced changes (Moll et al., 2021; Waters et al., 2016).

The time calls for embracing the responsibility in our functional role. Evolutionary changes in the life history of harvested populations and direct fishing impacts increase the probability of regime shifts within the ecosystems, and these might be hard to reverse (Conversi et al., 2015). It is, therefore, crucial to act sooner than later. Action and research are not mutually exclusive; thus, we encourage applicative studies on the impacts that FIE can trigger within marine food webs. In the meantime, we need to speculate based on incomplete knowledge and mitigate the contemporary consequences to prevent borrowing fish from future generations.

## CONFLICT OF INTEREST

We have no conflicts of interest to disclose.

## Data Availability

Data sharing not applicable to this article as no data sets were generated or analysed during the current study.
